# Novel Magnetically Recoverable Amino-Functionalized MIL-101(Fe) Composite with Enhanced Adsorption Capacity for Pb(II) and Cd(II) Ions

**DOI:** 10.3390/molecules30132879

**Published:** 2025-07-07

**Authors:** Claudia Maria Simonescu, Daniela C. Culita, Gabriela Marinescu, Irina Atkinson, Virgil Marinescu, Ovidiu Oprea, Nicolae Stanica

**Affiliations:** 1 Faculty of Chemical Engineering and Biotechnologies, National University of Science and Technology POLITEHNICA Bucharest, 313 Splaiul Independentei, 060042 Bucharest, Romania; claudiamaria_simonescu@yahoo.com (C.M.S.); ovidiu73@yahoo.com (O.O.); 2 Ilie Murgulescu Institute of Physical Chemistry, 202 Splaiul Independentei, 060021 Bucharest, Romania; gmarinescu@icf.ro (G.M.); iatkinson@icf.ro (I.A.); nstanica@icf.ro (N.S.); 3 National Institute for Research and Development in Electrical Engineering ICPE-CA, 313 Splaiul Unirii, 030138 Bucharest, Romania; marinescu_v@icpe-ca.ro; 4 Academy of Romanian Scientists, 3 Ilfov Street, 050044 Bucharest, Romania

**Keywords:** magnetite, NH_2_-MIL-101(Fe), lead, cadmium, adsorption

## Abstract

In this study, we report the synthesis and characterization of a novel NH_2_-MIL-101(Fe) magnetic composite, developed via in situ formation of NH_2_-MIL-101(Fe) in the presence of Fe_3_O_4_ nanoparticles embedded within a chloropropyl-modified mesoporous silica layer. This hybrid composite retains the high adsorption capacity of NH_2_-MIL-101(Fe) while benefiting from the easy magnetic separation enabled by Fe_3_O_4_ nanoparticles. The mesoporous silica forms a protective porous coating around the magnetic nanoparticles, significantly enhancing its chemical stability and preventing clumping. Beyond protection, the mesoporous silica layer provides a high-surface-area scaffold that promotes the uniform in situ growth of NH_2_-MIL-101(Fe). Functionalization of the silica surface with chloride groups enables strong electrostatic interactions between the magnetic component and metal organic framework (MOF), ensuring a homogeneous and stable hybrid structure. The new composite’s capacity to remove Pb(II) and Cd(II) ions from aqueous solutions was systematically investigated. The adsorption data showed a good fit with the Langmuir isotherm model for both ions, the maximum adsorption capacities calculated being 214.6 mg g^−1^ for Pb(II) and 181.6 mg g^−1^ Cd(II). Furthermore, the kinetic behavior of the adsorption process was accurately described by the pseudo-second-order model. These findings confirm the effectiveness of this composite for the removal of Pb(II) and Cd(II) ions from aqueous solutions, demonstrating its potential as an efficient material for environmental remediation. The combination of magnetic recovery, high adsorption capacity, and stability makes this novel composite a promising candidate for heavy metal removal applications in water treatment processes.

## 1. Introduction

Heavy metal ions are a class of pollutants that pose significant environmental and health risks due to their toxicity, persistence, and tendency to accumulate in living organisms. Common heavy metal pollutants include lead, mercury, cadmium, arsenic, chromium, and nickel. These metals are introduced into the environment through various activities such as mining, industrial processes, improper waste disposal, and the use of pesticides and fertilizers [1]. Unlike organic pollutants, heavy metal ions do not degrade or decompose in the environment. Instead, they persist and accumulate in soils, water, and the food chain, leading to long-term ecological and health consequences. Heavy metal contamination is a major concern because these ions can cause severe damage to living organisms, including neurological, cardiovascular, and developmental disorders in humans, as well as disrupting the normal functioning of ecosystems [2]. The persistence, bioaccumulation, and toxicity of heavy metal ions make them a significant environmental threat that requires urgent attention. Effective management and mitigation strategies are critical to minimizing their impact and protecting both human health and the environment. Various techniques have been developed and implemented to reduce heavy metal concentrations in contaminated environments, especially in water and soils. These methods can be categorized into physical (adsorption [3], membrane filtration [4], and electrodialysis [5]), chemical (chemical precipitation [6], ion exchange [7], and electrocoagulation [8]), and biological approaches (bioremediation [9] and phytoremediation [10]), each with specific advantages and limitations. Among these techniques, adsorption stands out as the most widely used method due to its high removal efficiency, simplicity, and low cost, especially when employing inexpensive and environmentally friendly materials [11]. A wide range of adsorbents, including activated carbon, zeolites, clays, nanoparticles, biomaterials, metal-organic frameworks, and metal oxides, have been explored for heavy metal ions removal from aqueous solutions [12].

Porous metal-organic frameworks (MOFs) are an advanced class of materials recognized for their exceptional ability to adsorb a wide range of pollutants (heavy metals, organic dyes, volatile organic compounds (VOCs), and greenhouse gases) due to their high surface area, tunable pore size, and customizable functionality [13,14]. These crystalline structures consist of metal ions or clusters coordinated to organic ligands, forming porous networks that can be engineered for specific adsorption tasks [15,16]. By modifying the metal nodes or organic linkers, MOFs can be tailored for the detection and selective adsorption of metal ions [17,18,19]. This selectivity is particularly valuable for treating water sources contaminated with multiple types of metal ions, allowing for targeted removal. Among various types of MOFs, MIL-101(Fe), a highly porous MOF composed of Fe(III) ions and terephthalic acid linkers, offers several advantageous properties, including a high surface area, large pore volume, and excellent thermal and chemical stability. Additionally, MIL-101(Fe) is environmentally friendly and cost-effective and can be regenerated for repeated use, making it a promising candidate for sustainable water purification and environmental remediation. These characteristics make it particularly suitable for capturing and sequestering heavy metal ions such as lead (Pb^2+^), cadmium (Cd^2+^), and chromium (Cr^6+^). NH_2_-MIL-101(Fe) is a functionalized variant of MIL-101(Fe) in which amino (-NH_2_) groups are introduced into the framework. This modification significantly enhances the material’s potential as adsorbent, offering additional active sites for binding heavy metal ions and improving selectivity and adsorption capacity. The textural properties and excellent stability of MIL-101(Fe) are retained, while the presence of -NH_2_ groups allows for stronger interactions, such as coordination and hydrogen bonding, with metal ions. These features make NH_2_-MIL-101(Fe) a highly effective material for removing toxic heavy metals from aqueous environments.

In practical applications, the separation and recovery of spent MOFs from aqueous solutions after the adsorption process can be challenging, mainly because MOFs are typically in powder form. To overcome this limitation, a common strategy is to incorporate MOFs into composite structures [20], such as magnetic composites [21,22,23,24] or gel-based composites [25,26]. The preparation of composites with other materials generates a synergistic effect that improves the adsorbent’s capacity, selectivity, and stability. Among these approaches, the development of Fe_3_O_4_-based magnetic MOF composites stands out due to the simplicity and convenience of the synthesis process. This is largely attributed to the use of magnetite (Fe_3_O_4_), which is inexpensive, stable, and easy to synthesize [27]. In recent years, Fe_3_O_4_-based magnetic MOF composites have been successfully fabricated via hydrothermal or solvothermal methods [28,29]. Two main strategies are typically employed for their synthesis: one involves the formation of core–shell structures, where the MOF shell is grown in situ on the Fe_3_O_4_ core surface, and the other relies on the direct attachment of Fe_3_O_4_ particles onto preformed MOFs through a one-step synthesis route.

Herein, we present a straightforward synthesis of a new NH_2_-MIL-101(Fe) magnetic composite through the in situ formation of NH_2_-MIL-101(Fe) in the presence of Fe_3_O_4_ nanoparticles embedded in a thin layer of chloropropyl-modified mesoporous silica, using a solvothermal method. The chloropropyl groups on the silica surface can provide reactive sites that facilitate the anchoring of NH_2_-MIL-101(Fe). These functional groups can engage in weak interactions with the amine groups of the MIL-101 framework, enhancing the attachment and stability of the metal-organic framework on the silica-coated magnetite nanoparticles. This ensures that NH_2_-MIL-101(Fe) grows uniformly on the silica-coated magnetite nanoparticles, preventing aggregation and allowing for controlled deposition of the MOF. The mesoporous silica layer also serves to protect the magnetite nanoparticles from potential oxidation. Magnetite is susceptible to degradation, which could compromise the magnetic properties required for separation; however, the functionalized silica layer acts as a protective barrier, maintaining the stability of the magnetic core. As part of our previous research studies aiming to develop new adsorbent materials for micropollutants existing in residual waters, we explored the adsorption properties of this new magnetic MOF composite for Pb(II) and Cd(II) ions. These metal ions were selected due to their high toxicity, environmental persistence, and frequent occurrence in industrial effluents. Both Pb(II) and Cd(II) are classified as priority pollutants by major environmental agencies due to their serious health and ecological risks. Therefore, studying their removal is both scientifically and environmentally relevant. Our findings demonstrate that integrating magnetic and porous components within a single architecture can significantly enhance the adsorption properties.

## 2. Results and Discussion

**Figure 1:**
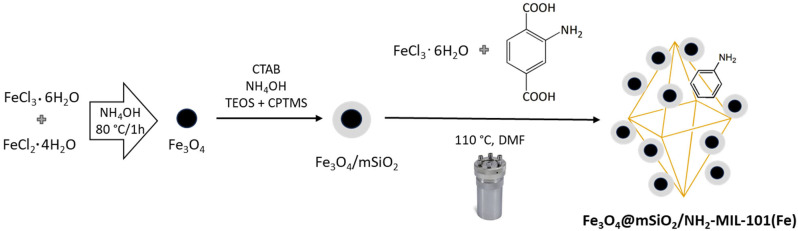
Schematic representation of the synthesis protocol for Fe_3_O_4_@mSiO_2_/NH_2_-MIL-101(Fe).

A schematic representation of the synthesis process for the magnetic composite Fe_3_O_4_@mSiO_2_/NH_2_-MIL-101(Fe) is shown in Figure 1, illustrating the main steps involved in the preparation procedure. Fe_3_O_4_ nanoparticles were first synthesized via coprecipitation of FeCl_3_·6H_2_O and FeCl_2_·4H_2_O in an alkaline medium, followed by coating with mesoporous silica. To obtain the final magnetic composite (Fe_3_O_4_@mSiO_2_/NH_2_-MIL-101(Fe)), Fe_3_O_4_@mSiO_2_ was dispersed in DMF and mixed with a solution containing FeCl_3_·4H_2_O and 2-aminoterephthalic acid, then subjected to solvothermal treatment in DMF at 110 °C, enabling the in situ growth of the NH_2_-MIL-101(Fe) framework on the surface of the Fe_3_O_4_@mSiO_2_ particles.

### 2.1. Characterization of the Materials

**Figure 2:**
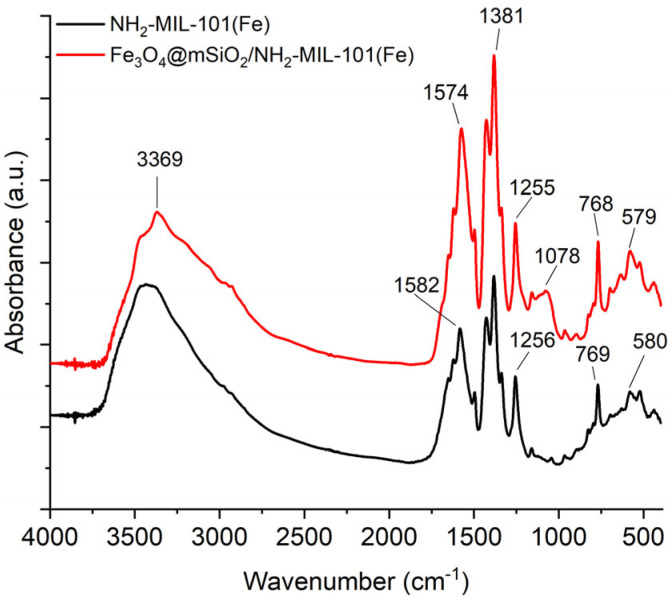
FT-IR spectra of Fe_3_O_4_@mSiO_2_/NH_2_-MIL-101(Fe) and NH_2_-MIL-101(Fe).

The Fourier transform infrared (FT-IR) spectra of Fe_3_O_4_@mSiO_2_/NH_2_-MIL-101(Fe) and NH_2_-MIL-101(Fe) are presented in Figure 2. The characteristic peaks of carboxylate groups belonging to the 2-amino-terephthalate ligands are identified at 1574 and 1381 cm^−1^ as the asymmetric and symmetric stretching vibration modes [30]. The band at 3369 cm^−1^ is assigned to the N-H stretching vibration, while that at 1255 cm^−1^ corresponds to the C-N stretching vibration [31]. The low-intensity absorption band at 1078 cm^−1^ can be attributed to the O-Si-O stretching vibration, confirming the presence of a silica coating on the Fe_3_O_4_ surface [32]. The peak at 579 cm^−1^ is assigned to the Fe-O stretching vibration [33].

**Figure 3:**
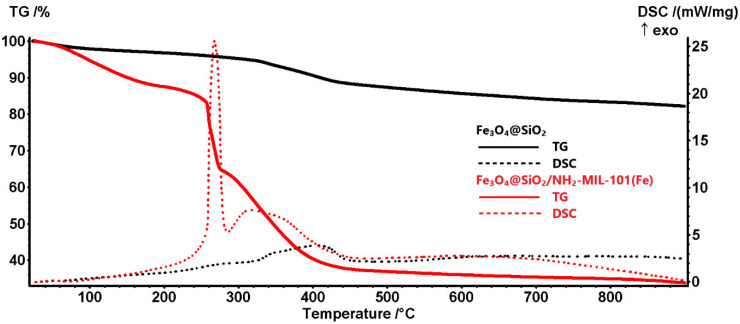
Thermal decomposition curves of Fe_3_O_4_@mSiO_2_/NH_2_-MIL-101(Fe) and Fe_3_O_4_@SiO_2_.

Thermogravimetric analysis coupled with differential scanning calorimetry (TG/DSC) was performed to evaluate the composition and thermal stability of Fe_3_O_4_@mSiO_2_/NH_2_-MIL-101(Fe). The analysis provides insights into the amount of NH_2_-MIL-101(Fe) formed on the surface of Fe_3_O_4_@mSiO_2_ and the degradation profile of the composite. Figure 3 presents the TG/DSC curves obtained. In the Fe_3_O_4_@mSiO_2_/NH_2_-MIL-101(Fe) sample, a 12.54% mass loss occurs up to 200 °C, associated with an endothermic process, with a minimum on the DSC curve at 72.6 °C. This mass loss can be attributed to the elimination of DMF molecules trapped in pores and residual humidity. Between 200 and 275 °C, a 22.49% mass loss was recorded. This process was highly energetic, with the majority of the mass loss occurring in the last 20 °C, accompanied by a strong and sharp exothermic effect at 268.1 °C. This suggests the oxidation of a flammable precursor or moiety, a behavior previously reported in the literature [34]. Between 275 and 500 °C, oxidative degradation of organic residues and combustion of carbonaceous mass occurs, leading to a 28.20% mass loss. This step is associated with a broad asymmetric exothermic effect, with a maximum at 317.5 °C. The residual mass at 900 °C was 33.78%.

The Fe_3_O_4_@mSiO_2_ sample loses 3.24% of its initial mass up to 200 °C, a process accompanied by a very weak endothermic effect at 57 °C. This step is most likely associated with the elimination of adsorbed water molecules. The main mass loss step (9.44%) occurs between 200 and 500 °C, with an exothermic effect displaying peaks at 277, 341, and 405.6 °C, suggesting multiple overlapping reactions. The exothermic nature of these effects indicates oxidation processes during which residual organic fragments are burned off from the silica shell. After 500 °C, the sample continues to lose mass due to densification and crystallization, originating from the condensation of surface -OH moieties and water elimination. The residual mass at 900 °C is 82.16%. When unprotected magnetite is heated in an oxidative atmosphere, Fe(II) oxidizes to Fe(III), transforming magnetite into maghemite below 300 °C [35]. This transformation is difficult to detect on TG curves, as the mass increase due to oxidation is often masked by concurrent mass losses from adsorbed water elimination and -OH moiety condensation. Similarly, on DSC curves, the exothermic effect is small and can be overshadowed by overall endothermic desorption processes or the exothermic oxidation of organic residues. However, the absence of the characteristic 500–600 °C peak for the maghemite-to-hematite transformation (γ-Fe_2_O_3_ → α-Fe_2_O_3_) [36] strongly confirms the protective role of the silica shell. Both residual samples exhibit strong magnetic properties and display the same reddish color. Based on the difference between the total weight loss of Fe_3_O_4_@mSiO_2_/NH_2_-MIL-101(Fe) (67.22%) and Fe_3_O_4_@mSiO_2_ (17.84%), we estimate that the NH_2_-MIL-101(Fe) content in the composite is approximately 50%.

**Figure 4:**
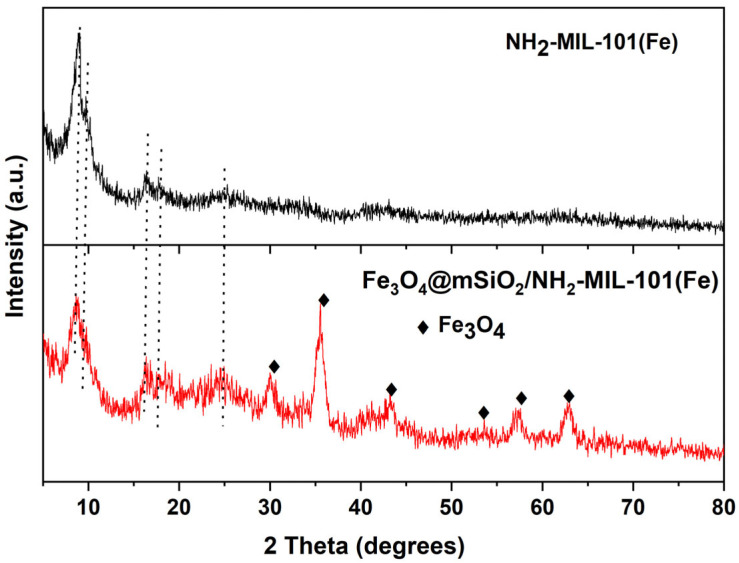
XRD patterns of Fe_3_O_4_@mSiO_2_/NH_2_-MIL-101(Fe) and NH_2_-MIL-101(Fe).

Figure 4 depicts the X-ray diffraction (XRD) patterns of NH_2_-MIL-101(Fe) and Fe_3_O_4_@mSiO_2_/NH_2_-MIL-101(Fe). The XRD pattern of the NH_2_-MIL-101(Fe) sample displays the diffraction peaks characteristic of NH_2_-MIL-101(Fe) structure at 2θ values of 9.01°, 10.1°, 16.23°, 17.9°, and 25.08°, consistent with those reported in the literature [37,38,39]. In the XRD pattern of the Fe_3_O_4_@mSiO_2_/NH_2_-MIL-101(Fe) composite, additional diffraction peaks appear at 2θ values of 30° (220), 35.5° (311), 43.2° (400), 53.3° (422), 57° (511), and 62.8° (440), corresponding to the cubic phase of magnetite (Fe_3_O_4_) and in good agreement with JCPDS No. 19-0629. Compared to the XRD pattern of pure NH_2_-MIL-101(Fe), the main peaks of NH_2_-MIL-101(Fe) are retained in the composite, showing only a slight decrease in crystallinity. The coexistence of diffraction peaks from both components in the XRD pattern confirms the presence of both the magnetic and MOF components in the composite.

**Figure 5:**
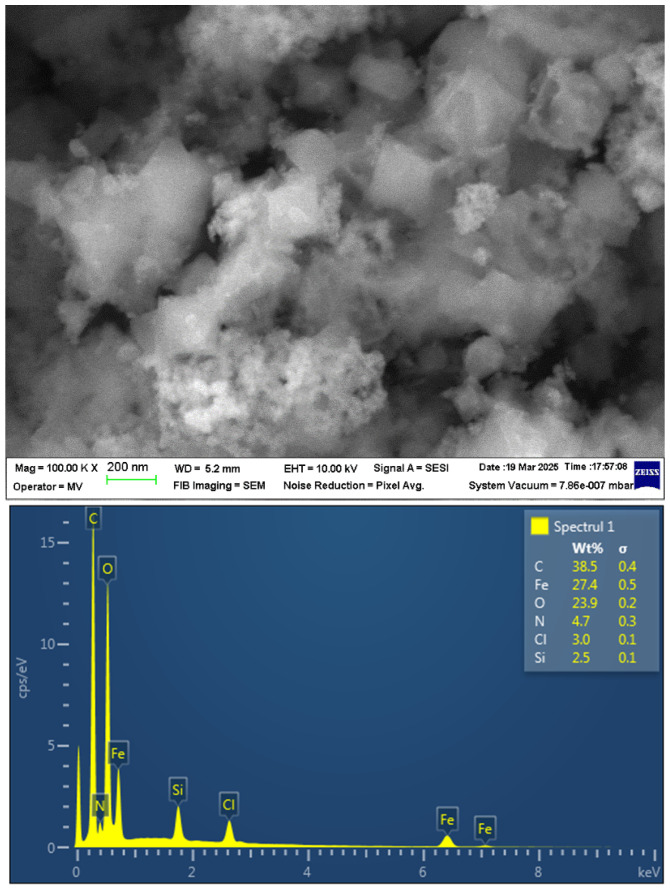
SEM image (top) and EDS spectrum (bottom) of Fe_3_O_4_@mSiO_2_/NH_2_-MIL-101(Fe).

The surface morphology of the Fe_3_O_4_@mSiO_2_/NH_2_-MIL-101(Fe) composite was investigated by scanning electron microscopy (SEM) analysis (Figure 5, top image). The SEM micrograph reveals a highly porous morphology composed of octahedral structures with smooth surfaces, characteristic of NH_2_-MIL-101(Fe), approximately 200–300 microns in size, embedded within a mesoporous silica network that encapsulates Fe_3_O_4_ nanoparticles. The elemental composition of Fe_3_O_4_@mSiO_2_/NH_2_-MIL-101(Fe) composite was further studied by energy dispersive spectroscopy (EDS). The EDS spectrum (Figure 5, bottom image) confirms the presence of C, Fe, O, N, Cl, and Si with weight percentages 38.5%, 27.4%, 23.9%, 4.7%, 3.0%, and 2.5%, respectively.

**Figure 6:**
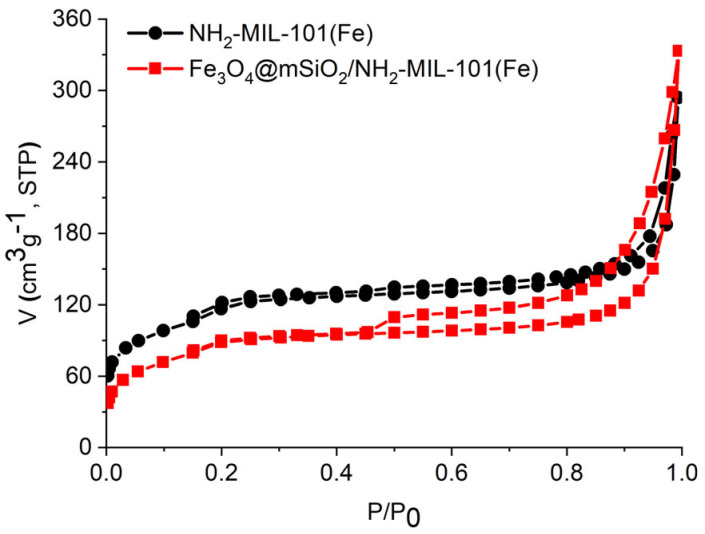
Nitrogen adsorption–desorption isotherms of Fe_3_O_4_@mSiO_2_/NH_2_-MIL-101(Fe) and NH_2_-MIL-101(Fe).

**Figure 7:**
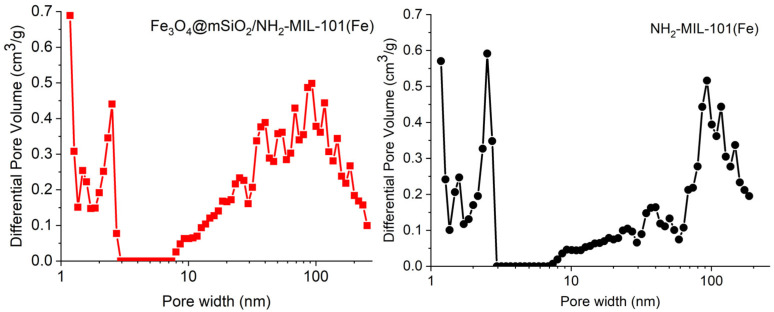
Pore size distribution curves Fe_3_O_4_@mSiO_2_/NH_2_-MIL-101(Fe) and NH_2_-MIL-101(Fe).

Figure 6 displays the nitrogen adsorption–desorption isotherms of the synthesized materials, Fe_3_O_4_@mSiO_2_/NH_2_-MIL-101(Fe) and NH_2_-MIL-101(Fe), which exhibit a combination of type I and IV [40], indicating that both materials contain micropores and mesopores. This isotherm profile reflects the material’s hierarchical pore structure. At low relative pressures, there is a steep increase in nitrogen uptake, indicative of micropore filling. This is followed by a plateau, reflecting multilayer adsorption on the sample surface. As pressure increases further (*p*/*p*_0_ > 0.4), capillary condensation occurs, indicating the presence of mesopores within the materials. The hysteresis loops of type H4 observable in the N_2_ isotherms further confirm the presence of mesopores [40]. As shown in Table 1, the specific surface area of Fe_3_O_4_@mSiO_2_/NH_2_-MIL-101(Fe) is lower than that of NH_2_-MIL-101(Fe) due to the partial pore blockage by the Fe_3_O_4_@mSiO_2_ particles. In any case, the surface area reduction is approximately 22%, a not very large value. The micropore surface area has the same proportion in both samples, approximately 8% of the total surface area. The shape of the Fe_3_O_4_@mSiO_2_/NH_2_-MIL-101(Fe) isotherm does not change substantially in comparison to NH_2_-MIL-101(Fe), which indicates that the introduction of the magnetic component does not affect the MOF formation. It is worth mentioning that the total pore volume for the sample containing magnetite is 13.4% higher than that of the NH_2_-MIL-101(Fe) sample, which is an advantage for an adsorbent material. Figure 7 illustrates the pore size distribution curves for Fe_3_O_4_@mSiO_2_/NH_2_-MIL-101(Fe) and NH_2_-MIL-101(Fe), showing quite similar distributions for the two materials, with a slightly higher mesopore volume for Fe_3_O_4_@mSiO_2_/NH_2_-MIL-101(Fe) compared to NH_2_-MIL-101(Fe).

**Figure 8:**
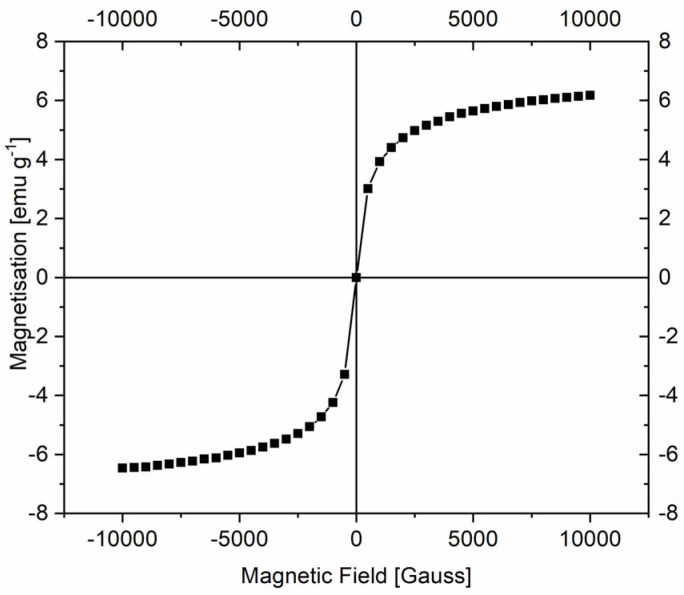
Magnetization curve of Fe_3_O_4_@mSiO_2_/NH_2_-MIL-101(Fe).

Given the importance of magnetic properties in adsorption applications, vibrating sample magnetometry (VSM) analysis was performed on the synthesized magnetic sample (Figure 8). The results show that Fe_3_O_4_@mSiO_2_/NH_2_-MIL-101(Fe) exhibits superparamagnetic behavior, which is generated by the magnetite contained in the sample, with a saturation magnetization of 5.34 emu/g. Despite this low value, which is attributed to the non-magnetic silica layer and NH_2_-MIL-101(Fe), the as-synthesized material allowed for the complete separation of particles under the influence of a permanent magnet.

### 2.2. Adsorption Studies

#### 2.2.1. Effect of pH and Adsorption Mechanism

**Figure 9:**
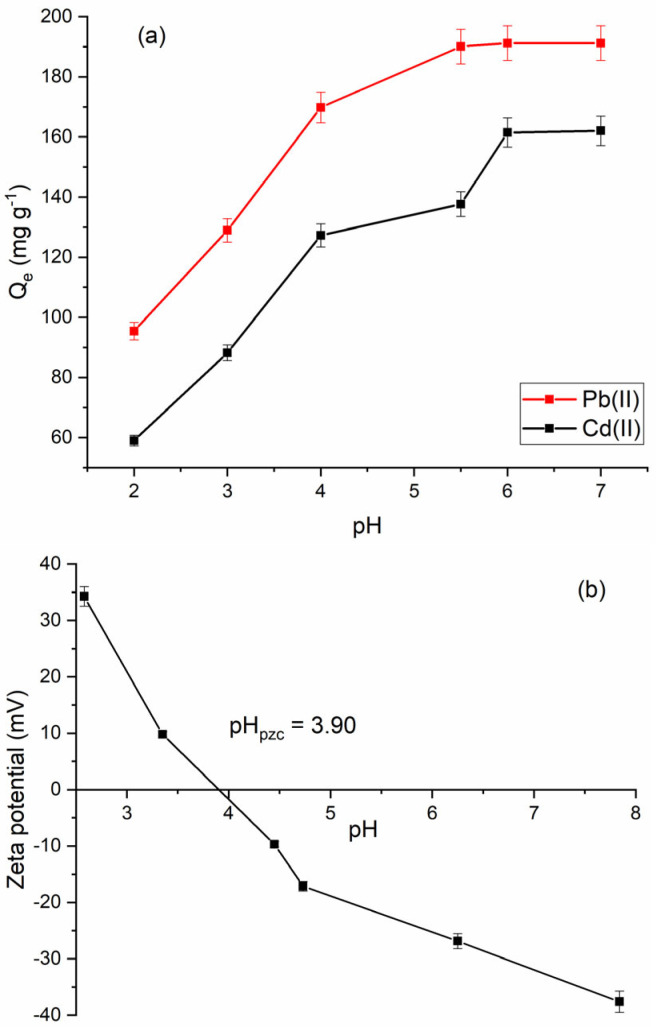
(**a**) Effect of the solution pH on the adsorption of Pb(II) and Cd(II) ions on Fe_3_O_4_@mSiO_2_/NH_2_-MIL-101(Fe); (**b**) zeta potential of Fe_3_O_4_@mSiO_2_/NH_2_-MIL-101(Fe) as a function of pH.

The effect of pH on the adsorption capacity of the material was studied in the range 2–6 for Pb(II) ions and 2–7 for Cd(II) ions. These pH ranges were selected to avoid the formation of insoluble hydroxide species such as Pb(OH)_2_ and Cd(OH)_2_, which could otherwise distort the interpretation of adsorption performance. As shown in Figure 9a, pH significantly influences the adsorption capacity of Fe_3_O_4_@mSiO_2_/NH_2_-MIL-101(Fe), with the highest adsorption capacities for Pb(II) and Cd(II) ions observed at higher pH values. At low pH, the adsorption capacity of the composite is significantly reduced. This can be attributed to the strong competition between the H^+^ and Pb^2+^/Cd^2+^ ions for the available active adsorption sites as well as the protonation of amino functional groups (-NH_2_) that hinders their coordination ability. As the pH increases to the range of 4–6, the adsorption of Pb^2+^/Cd^2+^ ions increases significantly. This behavior is supported by zeta potential measurements performed as a function of pH (Figure 9b). The surface of the Fe_3_O_4_@mSiO_2_/NH_2_-MIL-101(Fe) composite becomes increasingly negative with increasing pH. The point of zero charge (pH_pzc_) was determined to be 3.9, which is consistent with values reported in the literature for similar materials [41]. Below this value, the surface is positively charged, which limits the attraction of cationic species. Above the pH_pzc_, the surface acquires a negative charge, enhancing electrostatic interactions with Pb^2+^ and Cd^2+^ and thereby facilitating adsorption. In addition to electrostatic attraction, the -NH_2_ groups within the NH_2_-MIL-101(Fe) framework act as key adsorption sites, functioning as Lewis bases that can form coordination bonds with soft Lewis acid cations such as Pb^2+^ and Cd^2+^ ions. It is well known that the -NH_2_ groups have high affinity for heavy metal ions [42]. Moreover, the silanol (-Si-OH) groups present on the surface of mesoporous silica may also contribute to the adsorption process. These groups can interact with Pb^2+^ and Cd^2+^ through weak interactions, such as electrostatic attraction or weak van der Waals forces, particularly as the surface becomes more negatively charged at higher pH values. The porous structure of the composite also enhances adsorption by providing a high surface area and well-developed pore network that facilitates physical adsorption and diffusion of the metal ions into the framework. Overall, the adsorption of Pb^2+^ and Cd^2+^ onto Fe_3_O_4_@mSiO_2_/NH_2_-MIL-101(Fe) is the result of the synergistic contribution of various physicochemical interactions, involving mainly complexation by amino groups, weak interactions with hydroxyl groups of the mesoporous silica, and physical adsorption within the porous structure of the composite. Based on the observed high adsorption capacities, a pH of 6 was identified as optimal for both metal ions and used in further investigations.

#### 2.2.2. Equilibrium Adsorption Isotherms

Evaluating equilibrium data provides valuable insights into the interactions between the adsorbent and adsorbate as well as into the overall adsorption capacity of the adsorbent. The adsorption isotherm experiments were conducted at different initial concentrations of Pb(II) and Cd(II) ions to determine the maximum adsorption capacity of the studied material. The equilibrium data were analyzed using the Langmuir and Freundlich models. These models are mathematically expressed by the following equations:

(1) $$\mathrm{Langmuir}\;\mathrm{model}:\;Q_{e}=\frac{K_{L}Q_{m}C_{e}}{1+K_{L}C_{e}}$$

(2) Freundlich model: Qe=KF⋅Ce1n

where C_e_ (mg L^−1^) and Q_e_ (mg g^−1^) are the equilibrium concentration of adsorbate and the corresponding equilibrium adsorption capacity; Q_m_ (mg g^−1^) is the maximum adsorption capacity corresponding to complete monolayer coverage in the Langmuir model; K_L_ (L mg^−1^) is the Langmuir equilibrium constant associated with the adsorption energy; K_F_ and n are the Freundlich characteristic constants.

**Figure 10:**
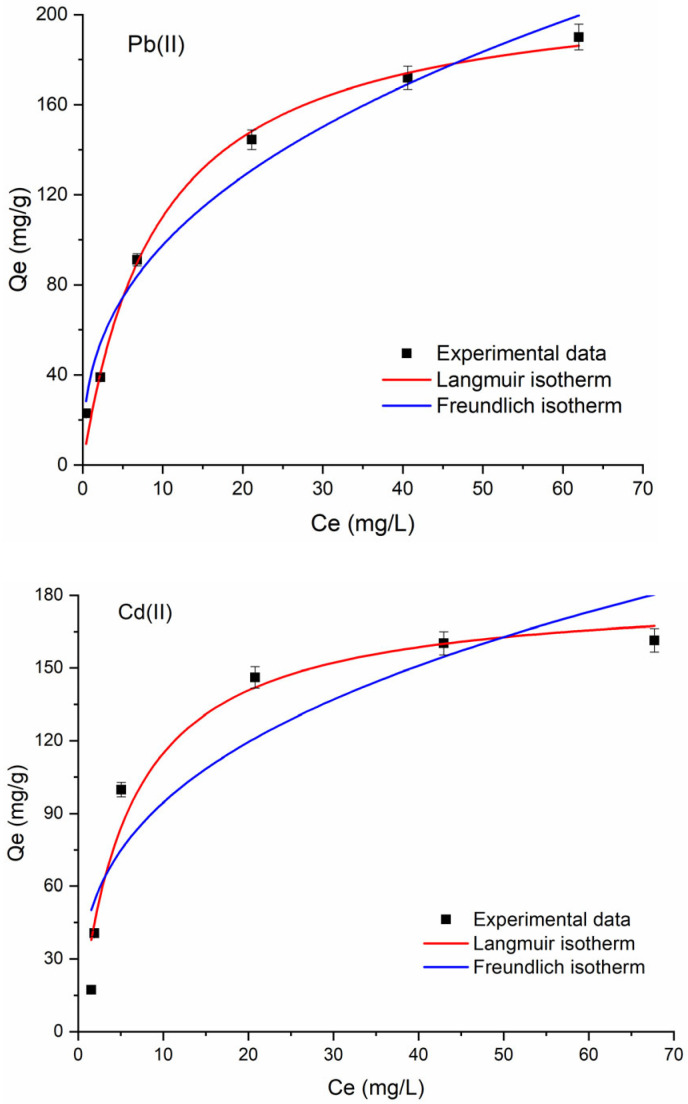
Equilibrium adsorption isotherms of Pb^2+^ and Cd^2+^ on Fe_3_O_4_@mSiO_2_/NH_2_-MIL-101(Fe).

The results are presented in Figure 10. They show that the equilibrium adsorption capacity for Pb(II) and Cd(II) ions increases significantly at low concentrations, then gradually rises until reaching the upper limit of the adsorption capacity of the adsorbent. The accuracy of model fittings to the experimental data was assessed using the regression coefficient (R^2^) and Akaike’s information criterion (AIC). A lower AIC value indicates a higher probability that the given model is the best fit compared to alternative models [43]. The R^2^ and AIC values (Table 2 and Table 3) indicate that the Langmuir model provided a better fit to the experimental data compared to the Freundlich model for both metal ions, suggesting that the adsorption is primarily monolayer adsorption on a relatively homogeneous surface. The estimated Qm values of Fe_3_O_4_@mSiO_2_/NH_2_-MIL-101(Fe) for Pb(II) and Cd(II) ions were 214.6 mg g^−1^ and 181.6 mg g^−1^, which are higher compared to some recently reported similar adsorbents (Table 4).

#### 2.2.3. Effect of Contact Time

**Figure 11:**
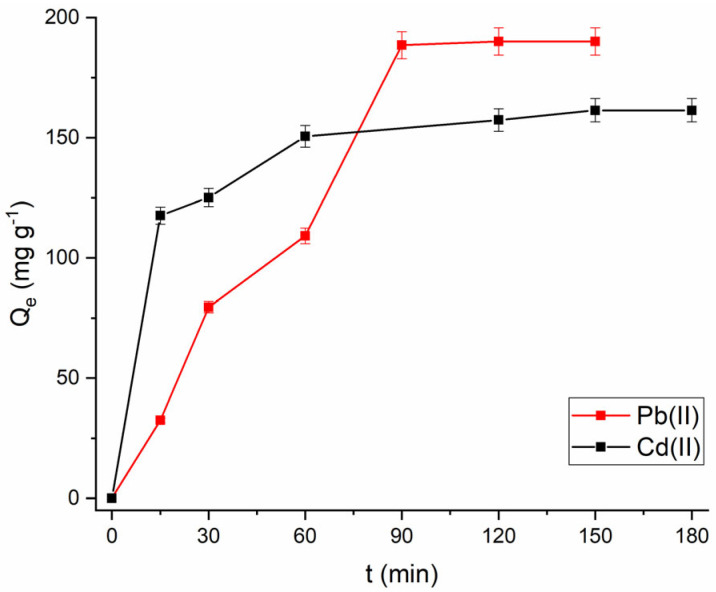
Effect of contact time on adsorption of Pb(II) and Cd(II) onto Fe_3_O_4_@mSiO_2_/NH_2_-MIL-101(Fe).

The influence of contact time on the adsorption process was investigated at a metal ion concentration of 100 mg L^−1^. As illustrated in Figure 11, the amount of ions adsorbed onto Fe_3_O_4_@mSiO_2_/NH_2_-MIL-101(Fe) increases with contact time, with the adsorption process being significantly rapid during the first 60 min. This behavior is attributed to the fast transport of metal ions into the adsorbent structure, which contains amino groups available for interaction, as well as the high solute concentration gradient. After this period, the adsorption rate slows down as the number of available amino groups decreases, and intraparticle diffusion becomes more limited. Equilibrium was reached after 120 min for Pb(II) and 150 min for Cd(II), indicating the saturation of all adsorption sites. The equilibrium values were 190.05 mg·g^−1^ for Pb(II) and 161.45 mg·g^−1^ for Cd(II), which are relatively close to the theoretical values. The adsorption capacity of the adsorbent for Pb(II) ions was higher than that for Cd(II) ions, likely due to differences in their affinities for NH_2_ groups. Pb(II) exhibits a stronger attraction to the lone electron pairs of amino nitrogen atoms than Cd(II) due to its higher absolute electronegativity, allowing it to form a stable complex more easily.

#### 2.2.4. Kinetic Analysis

**Figure 12:**
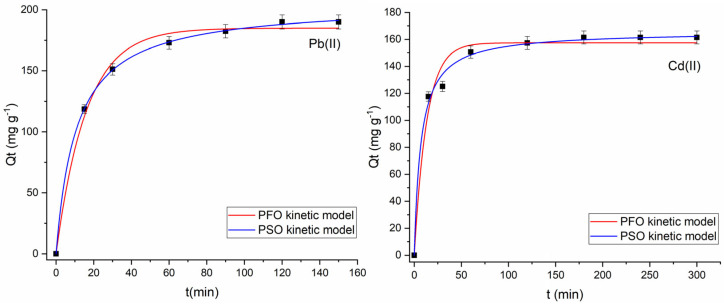
Graphical representation of the pseudo-first order (PFO) and pseudo-second order (PSO) kinetic models for Pb(II) and Cd(II) adsorption onto Fe_3_O_4_@mSiO_2_/NH_2_-MIL-101(Fe) (nonlinear regression).

To gain deeper insight into the adsorption process of Pb(II) and Cd(II) on Fe_3_O_4_@mSiO_2_/NH_2_-MIL-101(Fe) and to identify the rate-limiting steps, the kinetic data were analyzed using the non-linear forms of the pseudo-first-order (PFO) and pseudo-second-order (PSO) models (Figure 12). The pseudo-first-order model assumes that adsorption occurs mainly through physical interactions, while the pseudo-second-order model suggests that chemisorption predominates [51]. The calculated parameters for both models are presented in Table 5. Although the theoretically calculated Qe values from the PFO and PSO models align closely with the experimental data in the case of Cd(II) adsorption, the PSO model exhibits a higher R^2^ value and a lower AIC value. Therefore, the PSO model provides a more reliable description of the adsorption kinetics of Pb(II) and Cd(II) ions on Fe_3_O_4_@mSiO_2_/NH_2_-MIL-101(Fe), suggesting that the rate-limiting step involves chemisorption. These findings align with the proposed mechanism involving mainly coordination by amino groups and weak interactions with hydroxyl groups on the silica surface.

#### 2.2.5. Reusability Study

**Figure 13:**
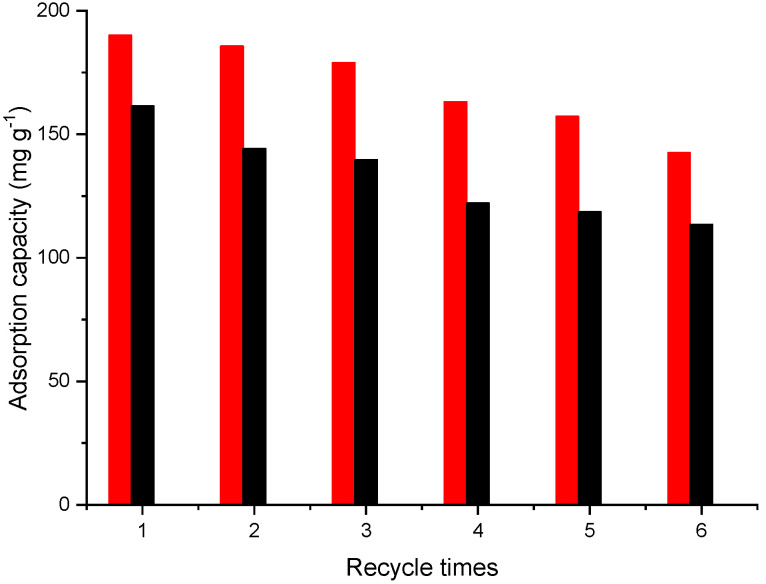
Reusability of Fe_3_O_4_@mSiO_2_/NH_2_-MIL-101(Fe) for Pb(II) (red) and Cd(II) (black).

The regeneration and reusability of an adsorbent are critical factors in assessing its practical viability for industrial applications. To evaluate the reusability of Fe_3_O_4_@mSiO_2_/NH_2_-MIL-101(Fe) for Pb(II) and Cd(II) adsorption, two desorption agents, 0.1 M HCl and 0.1 M HNO_3_, were tested in the regeneration process. The best results were achieved using 0.1 M HCl. Figure 13 presents the variation in adsorption capacity over six adsorption–desorption cycles. As shown, after six reuse cycles, the adsorption capacity of the adsorbent decreased to 75% for Pb(II) and 70% for Cd(II) compared to the initial values. This reduction can be attributed to several factors, including partial loss or deactivation of active adsorption sites during repeated regeneration steps and incomplete desorption of metal ions that may block adsorption sites in subsequent cycles. This relatively low reduction in adsorption capacity highlights the Fe_3_O_4_@mSiO_2_/NH_2_-MIL-101(Fe) composite as a highly effective and reusable material for the adsorption of Pb^2+^ and Cd^2+^ from contaminated water.

## 3. Materials and Methods

### 3.1. Materials

Ferric chloride hexahydrate > 99% (FeCl_3_·6H_2_O), ferrous chloride tetrahydrate > 98% (FeCl_2_·4H_2_O), cadmium nitrate tetrahydrate 98% (Cd(NO_3_)_2_·4H_2_O), lead(II) nitrate > 99.5% (Pb(NO_3_)_2_), ammonia solution (NH_4_OH 25 wt.%), tetraethoxysilane > 99% (TEOS), 3-(chloropropyl)-trimethoxysilane > 98%, ammonium nitrate > 95% (NH_4_NO_3_), ethanol absolute > 99.9%, methanol > 99.9%, dimethylformamide > 99.8% (DMF), hydrochloric acid (0.1 M), and nitric acid (0.1 M) were purchased from Merck (Darmstadt, Germany). 2-Amino-terephthalic acid 99% was purchased from Thermo Scientific, while cetyltrimethylammonium bromide ≥ 99% (CTAB) was from Carl Roth GmbH (Karlsruhe, Germany).

### 3.2. Synthesis of Fe_3_O_4_@mSiO_2_

The Fe_3_O_4_@mSiO_2_ particles were synthesized using a previously reported method with slight modifications [52]. In a typical procedure, an aqueous solution of 3.7 mmol FeCl_3_·6H_2_O and 1.84 mmol FeCl_2_·4H_2_O in 60 mL water was mixed under vigorous stirring with 15 mL of 25% NH_4_OH solution. This resulted in the immediate formation of a black precipitate. The mixture was heated at 80 °C for one hour, then the magnetic powder was separated and washed few times with distilled water until neutral pH. The freshly prepared magnetite particles were dispersed in a water/ethanol mixture (volume ratio 4:3) using ultrasonication. Next, 1.65 mmol CTAB and 1.2 mL of 25% NH_4_OH were added to the suspension and stirred at room temperature. After 1 h, 0.75 mL TEOS and 0.15 mL (3-chloropropyl)-trimethoxysilane (CTPMS) were added dropwise to the mixture, which was then stirred continuously at room temperature for 24 h. The resulting solid was collected with a permanent magnet and washed with hot methanol and deionized water multiple times. Finally, the template was removed by suspending the magnetic material in 150 mL ethanolic solution containing 3.75 mmol NH_4_NO_3_ and stirring for 1.5 h at 60 °C. The extraction process was repeated twice, and the final product, denoted as Fe_3_O_4_@mSiO_2_, was washed with ethanol and dried at 70 °C for 6 h.

### 3.3. Synthesis of Fe_3_O_4_@mSiO_2_/NH_2_-MIL-101(Fe)

Next, 0.1 g Fe_3_O_4_@mSiO_2_ was dispersed by ultrasonication in a 7.5 mL solution of DMF containing 2.5 mmol FeCl_3_·4H_2_O (solution A). In a separate beaker, 1.25 mmol 2-amino-terephthalic acid was dissolved in 7.5 mL DMF (solution B). The two solutions A and B were mixed, and the resulting mixture (solution C) was sonicated in an ultrasound bath for 15 min. Solution C was then poured into a Teflon-lined autoclave and treated at 110 °C for 20 h. After that, the precipitate was isolated by centrifugation, washed three times with DMF and three times with ethanol absolute, and dried at 80 °C for 12 h to obtain the Fe_3_O_4_@mSiO_2_/NH_2_-MIL-101(Fe) adsorbent.

For NH_2_-MIL-101(Fe), the synthesis steps were similar to those described for Fe_3_O_4_@mSiO_2_/NH_2_-MIL-101(Fe) excepting the addition of Fe_3_O_4_@mSiO_2._

### 3.4. Characterization Methods

FTIR spectra were collected using a Jasco FT/IR-4700 spectrophotometer (Jasco, Tokyo, Japan) in the 4000–400 cm^−1^ range, with samples pressed into KBr pellets. A Netzsch STA 449C Jupiter apparatus (Netzsch, Selb, Germany) was used for thermal analysis (TG-DSC). The samples were heated from room temperature to 900 °C at a rate of 10 °C/min in an open alumina crucible, under a flow of 20 mL/min dried air. An empty alumina crucible was used as a reference. Powder X-ray diffraction (XRD) patterns were obtained using a Rigaku Ultima IV diffractometer (Tokyo, Japan) with CuKα radiation (λ = 1.5406 Å) in the 2θ range of 10–80°, at a scanning speed of 5° min^−1^ and a step size of 0.02°, operating at 40 kV and 30 mA. SEM measurements were performed using a field emission scanning electron microscope (FESEM) workstation model Auriga (Carl Zeiss, Oberkochen, Germany), equipped with an Energy Dispersive Spectrometer (EDS INCA Energy 250 X-max 50, Oxford Instruments, Oxfordshire, UK). Nitrogen adsorption isotherms at −196 °C were recorded using a Micromeritics ASAP 2020 automated gas adsorption analyzer (Norcross, GA, USA). The samples were degassed under vacuum at 150 °C for 4 h prior to measurement. Specific surface areas (S_BET_) were calculated using the Brunauer–Emmett–Teller (BET) method. Total pore volume was estimated from the amount adsorbed at a relative pressure of 0.99. Pore size distributions were generated based on DFT calculations (slit–pore model). Zeta potential measurements were conducted using a Beckman Coulter Delsa Nano C analyzer (Brea, CA, USA) at 25 °C on samples suspended in distilled water (250 µg/mL). Magnetization measurements as a function of magnetic field were performed at room temperature using a Lake Shore Model 7404 Vibrating Sample Magnetometer (VSM) (Westerville, OH, USA).

### 3.5. Adsorption and Desorption Experiments

All adsorption experiments were performed in batch mode at room temperature, using a GFL 3031 incubating shaker. In the adsorption tests, stock solutions of 1000 mg/L Cd(II) and Pb(II) were prepared using Cd(NO_3_)_2_ and Pb(NO_3_)_2_. Working solutions with selected concentrations were prepared by diluting the stock solutions. To determine the optimum pH for adsorption, the initial pH of the Cd(II)/Pb(II) solutions was adjusted using NH_4_OH 10^−4^ mol/L and HNO_3_ 10^−3^ mol/L (analytical grade). The concentrations of Pb(II) and Cd(II) ions in the initial and treated solutions were measured with an AAnalyst 400 atomic absorption spectrometer (Perkin Elmer, Springfield, IL, USA). Standard solutions and samples solutions (Pb(II) and Cd(II)) were atomized with an air–acetylene flame (flame AAS) for electrothermal ionization. The instrumental parameters were as follows: for Pb(II)—wavelength: 283.3 nm, hollow-cathode lamp (lead), width of slit: 0.5 nm, and intensity of the lamp: 5 mA; and for Cd(II)—wavelength: 228.8 nm, hollow-cathode lamp (cadmium), width of slit: 1 nm, and intensity of the lamp: 3 mA. No background absorption correction was used. The pH of the solutions was determined with an Agilent 3200P laboratory pH meter. For each experiment, 5 mg of adsorbent material was added to 25 mL of Cd(II) or Pb(II) solution, each with an initial concentration of 100 mg/L. The solutions were stirred at 175 rpm while maintaining pH values in the range 2–7 for Cd(II) ions and 2–6 for Pb(II) ions. Suspensions were subsequently filtered, and the residual concentrations of Cd(II) and Pb(II) in the solutions were measured. The adsorption capacity (Q_e_, mg g^−1^) of the adsorbent and the amount adsorbed per unit mass of adsorbent at time t (Q_t_, mg g^−1^) were calculated using the following equations:

(3) $$Q_{e}=\frac{\left(C_{0}-C_{e}\right)\times V}{m}$$

(4) $$Q_{t}=\frac{\left(C_{0}-C_{t}\right)\times V}{m}$$

where C_0_ and C_e_ are the concentration of metal ion in the solution before and after adsorption, respectively (mg L^−1^); V is the volume of solution (L); and m is the mass of the adsorbent used (g).

To evaluate the reusability of the adsorbent, six consecutive adsorption–desorption cycles were performed. For each cycle, 5 mg of Pb(II) or Cd(II)-loaded Fe_3_O_4_@mSiO_2_/NH_2_-MIL-101(Fe) was placed in 25 mL of 0.1 M HCl or 0.1 M HNO_3_ solution and stirred at room temperature for 150 min to desorb the retained metal ions. After desorption, the adsorbent was separated, thoroughly washed with deionized water to remove residual acid and metal ions, and then dried at 60 °C for 12 h before reuse. The adsorption capacity was measured after each cycle under the same initial adsorption conditions (pH, concentration, and contact time) to assess the stability and regeneration efficiency of the adsorbent over multiple cycles.

## 4. Conclusions

This study reports the synthesis and characterization of a novel magnetic composite, Fe_3_O_4_@mSiO_2_/NH_2_-MIL-101(Fe), developed by integrating magnetite nanoparticles embedded within a chloropropyl-modified mesoporous silica layer with amino-functionalized MIL-101(Fe) for the efficient removal of Pb(II) and Cd(II) ions from aqueous solutions. The composite combines high adsorption capacity with facile magnetic separation, offering a practical and versatile solution for wastewater treatment. Notably, it retains the high surface area, large pore volume, and structural stability characteristic of MIL-101(Fe), while gaining the added advantage of magnetic responsiveness. This dual functionality significantly enhances both performance and operational convenience. Kinetic studies revealed that the adsorption of both metal ions follows a pseudo-second-order model, indicating chemisorption as the dominant mechanism. The equilibrium adsorption data were best described by the Langmuir isotherm model, with maximum adsorption capacities of 214.6 mg g^−1^ for Pb(II) and 181.6 mg g^−1^ for Cd(II), values that exceed those of many similar materials reported in the literature. The adsorption mechanism analysis indicated that the amino groups in the NH_2_-MIL-101(Fe) framework play a key role in metal ion binding through coordination. Additionally, silanol groups from the mesoporous silica as well as the porous structure of the composite contribute to metal ions adsorption through electrostatic interactions and physical entrapment, respectively. These combined interactions confirm that the adsorption mechanism involves a synergistic effect of chemisorption, electrostatic attraction, and physical adsorption. Moreover, the new composite demonstrated good regeneration potential and recyclability. These findings highlight the composite’s potential as an effective, reusable, and scalable material for wastewater treatment applications.

## Figures and Tables

**Table 1:** Textural properties of the samples.

Sample	S_BET_ (m^2^/g)	S_micro_ (m^2^/g)	Pore Volume (cm^3^/g)
Fe_3_O_4_@mSiO_2_/NH_2_-MIL-101(Fe)	324.4	26.7	0.515
NH_2_-MIL-101(Fe)	418.5	33.8	0.454

**Table 2:** Langmuir isotherm parameters obtained by nonlinear regression.

	Langmuir Parameters				
	Q_m_ (mg g^−1^)	K_L_ (L mg^−1^)	R^2^	AIC	R_L_
Pb^2+^	214.6	0.1055	0.9887	39.6358	0.0865
Cd^2+^	181.6	0.1723	0.9541	46.7816	0.0548

**Table 3:** Freundlich isotherm parameters obtained by nonlinear regression.

	Freundlich Parameters			
	K_F_ (mg g^−1^)	1/n	R^2^	AIC
Pb^2+^	50.2054	0.3328	0.9483	45.5727
Cd^2+^	43.4725	0.3374	0.8175	55.0602

**Table 4:** Comparison of adsorption capacity of various MOF adsorbents for Pb(II) and Cd(II) ions.

Adsorbent	Adsorption Capacity for Pb(II) (mg g^−1^)	Adsorption Capacity for Cd(II) (mg g^−1^)	Ref.
Fe–MIL–101	86.20	-	[44]
MIL-101	57.96	-	[44]
ED-MIL-101(5 mmol)	81.09	-	[45]
ED-MIL-101(Cr)		63.15	[42]
SS-SO_3_H-MIL-101(Cr)-3	183.4	98.7	[46]
NH_2_-mSiO_2_@MIL-101(Cr)	161.3	-	[47]
UiO-66-NH_2_	135.0	-	[48]
CMOF-199	-	92.4	[49]
TMU-16-NH2	-	126.6	[50]
Fe_3_O_4_@mSiO_2_/NH_2_-MIL-101(Fe)	214.6	181.6	This work

**Table 5:** The kinetic parameters for Pb(II) and Cd(II) adsorption onto Fe_3_O_4_@mSiO_2_/NH_2_-MIL-101(Fe) (nonlinear regression).

Sample	Pb(II)	Cd(II)
Q_e_ exp (mg·g^−1^)	190.05	161.45
**Pseudo-first-order model**		
Q_e_ cal (mg·g^−1^)	185.01 ± 3.19	157.46 ± 3.90
k_1_ (min^−1^)	0.0624 ± 0.0050	0.0753 ± 0.0106
R^2^_adjusted_	0.9922	0.9753
AIC	36.76	44.20
**Pseudo-second-order model**		
Q_e_ cal (mg·g^−1^)	205.11 ± 0.99	165.99 ± 2.62
k_2_ (10^−4^ g·mg^−1^·min^−1^)	4.4839 ± 1.3909	8.4634 ± 1.1834
R^2^_adjusted_	0.9997	0.9936
AIC	13.47	33.38

## Data Availability

Data are contained within the article.
